# Modeling of human factor Va inactivation by activated protein C

**DOI:** 10.1186/1752-0509-6-45

**Published:** 2012-05-20

**Authors:** Maria Cristina Bravo, Thomas Orfeo, Kenneth G Mann, Stephen J Everse

**Affiliations:** 1 Cell and Molecular Biology Program, University of Vermont, 89 Beaumont Ave, Burlington, VT 05405, USA; 2 Department of Biochemistry, University of Vermont, 208 South Park Drive, Colchester, VT 05446, USA; 3 Department of Biochemistry, University of Vermont, 89 Beaumont Ave., Given B418, Burlington, VT 05405, USA

**Keywords:** Coagulation, Factor Va, Activated protein C, *Prothrombinase*, Prothrombin, Factor Xa, Mathematical modeling

## Abstract

**Background:**

Because understanding of the inventory, connectivity and dynamics of the components characterizing the process of coagulation is relatively mature, it has become an attractive target for physiochemical modeling. Such models can potentially improve the design of therapeutics. The *prothrombinase* complex (composed of the protease factor (F)Xa and its cofactor FVa) plays a central role in this network as the main producer of thrombin, which catalyses both the activation of platelets and the conversion of fibrinogen to fibrin, the main substances of a clot. A key negative feedback loop that prevents clot propagation beyond the site of injury is the thrombin-dependent generation of activated protein C (APC), an enzyme that inactivates FVa, thus neutralizing the *prothrombinase* complex. APC inactivation of FVa is complex, involving the production of partially active intermediates and “protection” of FVa from APC by both FXa and prothrombin. An empirically validated mathematical model of this process would be useful in advancing the predictive capacity of comprehensive models of coagulation.

**Results:**

A model of human APC inactivation of *prothrombinase* was constructed in a stepwise fashion by analyzing time courses of FVa inactivation in empirical reaction systems with increasing number of interacting components and generating corresponding model constructs of each reaction system. Reaction mechanisms, rate constants and equilibrium constants informing these model constructs were initially derived from various research groups reporting on APC inactivation of FVa in isolation, or in the presence of FXa or prothrombin. Model predictions were assessed against empirical data measuring the appearance and disappearance of multiple FVa degradation intermediates as well as *prothrombinase* activity changes, with plasma proteins derived from multiple preparations. Our work integrates previously published findings and through the cooperative analysis of *in vitro* experiments and mathematical constructs we are able to produce a final validated model that includes 24 chemical reactions and interactions with 14 unique rate constants which describe the flux in concentrations of 24 species.

**Conclusion:**

This study highlights the complexity of the inactivation process and provides a module of equations describing the Protein C pathway that can be integrated into existing comprehensive mathematical models describing tissue factor initiated coagulation.

## Background

**Figure 1:**
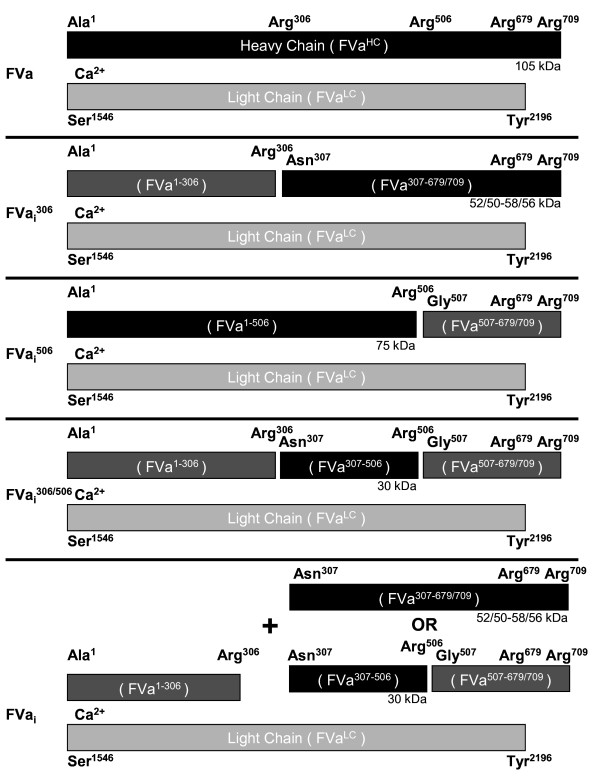
**Schematic Representation of Factor Va and the Mechanism of the APC-Generated Partially Proteolyzed Species.** Human factor Va (FVa), consisting of the non-covalently associated 105 kDa heavy chain (FVa^HC^) and 74/72 kDa light chain (two carbohydrate variants) (FVa^LC^), has maximal cofactor activity in the *prothrombinase* complex. FVa contains three APC cleavage sites: Arg^306^, Arg^506^, and Arg^679^; cleavage at Arg^679^ plays a minimal role in altering cofactor activity and so will not be considered further. APC cleaves FVa in a random, non-sequential manner at Arg^306^ and Arg^506^ leading to the formation of partially proteolyzed species (FVa_i_^306^ and FVa_i_^506^, respectively). FVa_i_^306^ and FVa_i_^506^ are subsequently proteolyzed by APC at Arg^506^ or Arg^306^, respectively, to generate the FVa_i_^306/506^ species. *Prothrombinase* activity is possible even when the FVa^HC^ has been proteolyzed at both sites as long as the heavy chain fragments remain associated with each other and the FVa^LC^. The partially active FVa_i_^306^ and FVa_i_^306/506^ species lose full activity with the spontaneous dissociation of either the FVa^307-679/709^ or FVa^307-506^FVa^507-679/709^ fragments, respectively. Fragments shaded in black are recognized by the monoclonal antibody (αHFV#17) and their apparent (SDS-PAGE) molecular weights are indicated.

One of the critical events in blood coagulation is the conversion of large amounts of the zymogen prothrombin to the enzyme thrombin by the enzymatic complex *prothrombinase*. The *prothrombinase* complex is formed by the non-covalent interaction between the enzyme factor Xa (FXa) and the non-enzymatic cofactor factor Va (FVa) on a phospholipid surface in the presence of calcium [1-3]. The procofactor, factor V, is activated by thrombin to generate a calcium-associated two chain molecule, composed of a heavy chain (FVa^HC^) and light chain (FVa^LC^) (Figure 1) [4,5]. The *prothrombinase* complex increases thrombin generation over FXa alone by 5 orders of magnitude [2]. The explosive burst in prothrombin conversion is essential to rapidly form a stable fibrin clot in response to a vascular injury. The fibrin clot is formed when thrombin cleaves fibrinogen to form an insoluble polymer network [6,7]. This fibrin network becomes cross-linked and stabilized by factor XIIIa which also traps platelets and red blood cells sealing the wound [7-9].

It is critical that once a procoagulant response to vascular injury has been initiated that an appropriate anticoagulant response is concurrently mounted. As such, the components of the *prothrombinase* complex are targets of multiple regulatory mechanisms to terminate the production of thrombin. First, FXa availability is regulated by formation of inhibition complexes with antithrombin (AT), α_1_-antitrypsin, and tissue factor pathway inhibitor (TFPI) [10,11]. The cofactor, FVa, is a target for degradation by activated protein C (APC). APC, a serine protease derived from its plasma precursor protein C (PC) in a thrombin dependent process [12], plays a critical role in inactivating the non-enzymatic cofactor components of both the *prothrombinase* and the *intrinsic* tenase complexes, factors Va and VIIIa, respectively [13-15]. Additional key components of the PC pathway include: thrombomodulin and endothelial protein C receptor (EPCR) which contribute to APC formation [16]; protein S which functions as a cofactor enhancing APC efficacy [17]; and protein C inhibitor, a suppressor of APC formation [18,19].

An increased risk of thrombotic disease has been associated with partial deficiencies or loss of function mutations in the PC pathway, including deficiencies in PC, its cofactor protein S, or proteins involved in the activation of PC [20]. The most prevalent defect in the PC pathway is a result of a genetic mutation in FV that renders the cofactor resistant to APC inactivation [21]. This resistance to APC was first characterized by Dahlbäck and coworkers [21] and derives from a mutation at one of the APC inactivation sites (Arg^506^ → Gln^506^) on FV/FVa rendering it resistant to APC cleavage [22].

APC catalyzed inactivation of FVa cofactor activity is a complex, membrane dependent process involving cleavage at multiple sites in the FVa^HC^, the generation of transient species with partial cofactor activity, and the ultimate disassociation of a fragment of the FVa^HC^ from the molecule rendering it catalytically inactive. APC interacts with FVa or partially proteolyzed FVa species through their light chains to form enzymesubstrate complexes [23-25]. Additional studies have identified other regions of interaction, including the proteolytic target residues and the surrounding regions, in the FVa heavy chain involved in the enzymesubstrate complex formation [26-28]. Three arginine residues are targeted in human APCFVa complexes: Arg^306^, Arg^506^, and Arg^679^[15,29]. A number of studies have defined the activities of the partially proteolyzed FVa species [30,31]. Cleavage at either Arg^306^ or Arg^506^ results in cofactor species, FVa_i_^306^ and FVa_i_^506^, respectively, with reduced but similar cofactor activity in the *prothrombinase* complex (Figure 1) [30,31]. Cleavage at Arg^306^ has been shown to be essential for full loss of cofactor activity [32]. The significance of the cleavage at Arg^679^ remains undetermined. The final inactive cofactor, FVa_i_, is a two chain molecule composed of the FVa^LC^ and FVa^1-306^ fragment (Figure 1) [32]. The inactive cofactor binds APC with the same affinity as the intact cofactor [23,33], suggesting a potential role of product inhibition in regulating APC efficacy [33].

**Figure 2:**
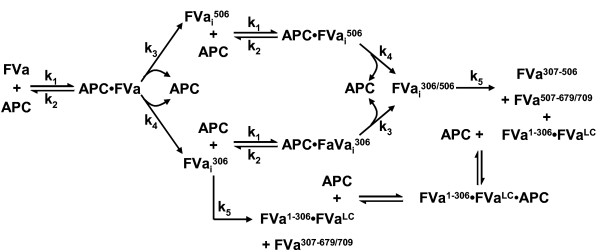
**Mechanism of the APC Inactivation of Factor Va.** Random non-ordered APC cleavage mechanism of bovine FVa proposed by Hockin et al. [33] renumbered with human FVa cleavages.

The complexity of this process led Hockin and coworkers to construct an ordinary differential equation based description of the network of reactions characterizing the process of bovine APC inactivation of bovine FVa using rate constants gleaned from studies with bovine, human and recombinant human proteins [33]. Empirical studies from a number of laboratories detailing APC inactivation of FVa have shown the cleavage at Arg^506^ preceding that at Arg^306^, with these data initially (often) being interpreted as indicating a sequential or ordered kinetic mechanism [15]. However, based on their mathematical modeling, Hockin et al. [33], concluded that the reaction pathway was better described as a random order cleavage process for Arg^306^ and Arg^505^ (Arg^506^ in human FVa) in which each of the cleavages is independent of the other, but occurs at different rates (Figure 2), and where full loss of cofactor activity requires disassociation of the fragment of the FVa^HC^ downstream from the Arg^306^ cleavage [32,33].

Numerous studies examining how components like FXa, prothrombin and protein S modulate APC inactivation of FVa have been reported [14,34-40]. These studies routinely employ approaches like progress curve analysis based on curve fitting to compare initial rates, or natural and recombinant cleavage site mutants to detail mechanistic features. To date, however, an integrated approach, one combining the use of physicochemical model construction based on prior research and model verification via construction of corresponding reaction systems using purified proteins, has not been extended to understand APC regulation of the *prothrombinase* complex stability and function. In this study we build on the prior studies modeling APC inactivation of FVa [33] by incorporating the presence of additional components of the *prothrombinase* complex (FXa and prothrombin) to construct an empirically validated mathematical model. To test model constructs, a step-wise approach to increasing the number of components was used. Experiments were replicated multiple times with different preparations of proteins in order to generate robust data sets that included estimates of measurement error. At each level of complexity, we empirically monitored multiple analytes, including starting reactants, intermediates and final products to provide multiple points of comparison to evaluate each model’s performance.

## Results and Discussion

### APC Inactivation of Factor Va

#### Empirical Analyses

**Figure 3:**
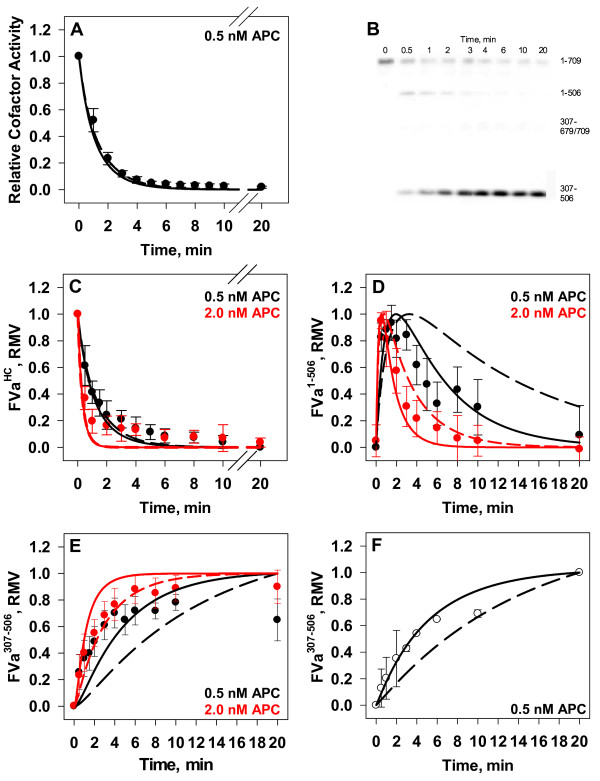
**Inactivation of Factor Va by Activated Protein C.** Empirical: FVa (20 nM, Panels *A-E*) or FVa_i_^506^ (20 nM, Panel *F*) was incubated with PC:PS vesicles (20 μM) and APC (0.5 or 2.0 nM) at 37°C. All data points are presented as averages ± S.D. of 4–6 experiments for each time point. (*A*) Time course of APC (0.5 nM) inactivation of FVa. Cofactor activity is presented relative to activity present at zero point (black circles). (*B*) Representative Western blot of time course of APC (2.0 nM) inactivation of FVa. (*C*) Densitometric analyses of the disappearance of FVa^HC^ at 0.5 or 2.0 nM APC (black and red circles, respectively). (*D*) Densitometric analyses of the time course of FVa^1-506^ at 0.5 or 2.0 nM APC (black and red circles, respectively). (*E*) Densitometric analyses of the time course of FVa^307-506^ at 0.5 or 2.0 nM APC (black and red circles, respectively). (*F*) Densitometric analyses of the inactivation fragment FVa^307-506^ generated during the APC (0.5nM) inactivation of FVa_i_^506^ (open circles). Inset displays a representative Western blot. Mathematical: Mathematically derived time courses of FVa inactivation by APC (0.5 nM - black lines and 2.0 nM - red lines) for the corresponding analytes are also presented in Panels A and C-F. Simulated time courses were generated using two sets of rate constants: Hockin et al. [33] – dashed lines; and current study – solid lines.

The inactivation of FVa (20 nM) was monitored at two APC concentrations (0.5 and 2.0 nM) in the presence of excess PC:PS vesicles (20 μM). The lower concentration of APC was used to initiate a slow inactivation reaction that would enable multiple time points to monitor the sequence of proteolytic events and the residual cofactor activity (as measured by a one stage clotting assay). In studies with 0.5 nM APC, within one minute there was a 50 % loss in cofactor activity (Figure 3, Panel A, black circles) which continued in a monophasic decay. According to previous studies, measurement of FVa cofactor activity as measured in clotting assays is sensitive to APC cleavage of Arg^506^[41].

Western blot analyses (representative blot in Figure 3, Panel B) monitoring the disappearance of intact FVa^HC^ (residues 1–709) display the rapid proteolysis of FVa heavy chain proteolyzed at either site within the first or third minutes with 2.0 or 0.5 nM APC, respectively (Figure 3, Panel C, red and black circles, respectively). These results were each fit to monophasic exponential decays (fit not shown), and the initial slope of the fitted curve was determined after normalizing the densitometry to the maximal value (RMV). At 0.5 nM APC, the initial rate of disappearance of the heavy chain was 200 pM/s (based on 20 nM starting concentration); at 2.0 nM APC, the rate of disappearance was nearly three-fold faster (558 pM/s). The disappearance of the heavy chain is initially concurrent with accumulation of the FVa^1-506^ fragment (Figure 3, Panel D). The generation of the FVa^1-506^ fragment reaches a maximum within 30 sec (2.0 nM APC) or 90 sec (0.5 nM APC) (Figure 3, Panel D, red and black circles, respectively). At this point most of the FVa^HC^ has been proteolyzed primarily at Arg^506^, and the secondary cleavage at Arg^306^ in the FVa^1-506^ species becomes the dominant proteolytic event. Analysis of the appearance of the inactivation fragment FVa^307-506^ (resulting from cleavage at both Arg^306^ and Arg^506^, Figure 3, Panel E), indicates that as the FVa^1-506^ fragment is approaching its maximum level it is being further proteolyzed to the FVa^307-506^ fragment. In order to isolate the kinetics of proteolysis at Arg^306^, FVa_i_^506^ was used as the starting material in inactivation studies with 0.5 nM APC (Figure 3, Panel F, open circles). This membrane bound partially proteolyzed species has already lost most of its cofactor activity in a clotting assay [30,31,33]. Tracking of the final inactivation fragment, FVa^307-506^, by Western blotting reveals a monophasic accumulation of this fragment (fit not shown) (Figure 3, Panel F).

#### Mathematical Simulations

Using the non-sequential reaction mechanism proposed by Hockin et al. (Table 1, Figure 2) [33] and kinetic constants used to describe their bovine system of proteins, *in silico* simulations of APC inactivation of FVa were performed and predicted time courses for various FVa inactivation species compared to the empirical data presented in Figure 3. Mathematical simulations show good correlation with empirical measurements of FVa cofactor activity and the densitometric analysis of FVa^HC^ (Figure 3, Panel A and C, dashed lines vs. filled circles, respectively). Hockin et al. reported a good fit using this model between simulated and empirical time courses of APC catalyzed changes in cofactor activity in the bovine system [33]. The present study’s correlation between clotting assay measurements, *in silico* FVa cofactor activity, and densitometric analysis of FVa^HC^ are consistent with the findings of earlier studies that FVa cofactor activity is based on the amount of intact FVa^HC^ present [41].

**Table 1:** Equations describing APC inactivation of factor Va

**Eqn**	**Chemical Expression**^**a**^	**k**_**off**_	**k**_**on**_	**k**_**cat**_	**k**_**dis**_
		**(s**^**-1**^**)**	**(M**^**-1**^ **s**^**-1**^**)**	**(s**^**-1**^**)**	**(s**^**-1**^**)**
1	APC + FVa ← 2-1 → APCFVa	0.7	1 x 10^8^		
2	APCFVa −3 → APC + FVa_i_^506^			1.0	
3	APCFVa −4 → APC + FVa_i_^306^			0.064^b^	
				0.192^c^	
4	APC + FVa_i_^506^ ← 2-1 → APCFVa_i_^506^	0.7	1 x 10^8^		
5	APC + FVa_i_^306^ ← 2-1 → APCFVa_i_^306^	0.7	1 x 10^8^		
6	APCFVa_i_^506^ -4 → APC + FVa_i_^306/506^			0.064^b^	
				0.192^c^	
7	APCFVa_i_^306^ -3 → APC + FVa_i_^306/506^			1.0	
8	FVa_i_^306^ -5 → FVa^1-306^FVa^LC^ + FVa^307-679/709^				0.028
9	FVa_i_^306/506^ -5 → FVa^1-306^FVa^LC^ + FVa^307-506^ + FVa^507-679/709^				0.028
10	APC + FVa^1-306^FVa^LC^ ← 2-1 → APC FVa^1-306^FVa^LC^	0.7	1 x 10^8^		

^a^ For equilibrium expressions denoted by ← 2-1→, the first number listed describes the reverse reaction given in the k_off_ column.

^b^ Rate constant used for mathematical simulations as determined by inactivation studies with bovine FVa and bovine APC in Hockin et al. [33].

^c^ Rate constant used for mathematical simulations, as determined by inactivation studies with human FVa and human APC in this study, is 0.192 s^-1^. This new rate constant will be used in all subsequent mathematical simulations.

However, the predicted generation and disappearance of the FVa^1-506^ fragment are significantly delayed compared to the empirical data for this inactivation fragment (Figure 3, Panel D, black dashed line vs. black circles). At 0.5 nM APC, the simulation predicts that this fragment will reach a maximum at 3.4 min before slowly disappearing, with 30 % still remaining after 20 min; the empirical experiments show the peak level reached at 1.5 min with only 9 % remaining after 20 min (Figure 3, Panel D, black dashed line vs. black circles). The mathematical simulation fared better with 2.0 nM APC (Figure 3, Panel D, red dashed line vs. red circles) with respect to the timing of peak accumulation, however failed to recapitulate the rapid clearance of this fragment. Mathematical simulations of FVa^307-506^ using the Hockin et al. [33] rate constants resulted in an estimated six-fold slower accumulation of this fragment compared to empirical data (Figure 3, Panel E, dashed lines vs. circles).

Though the bovine based Hockin et al. [33] rate constants clearly captured the disappearance of FVa^HC^ as measured through activity and fragment analysis, they did not accurately predict the presentation of either the FVa^1-506^ or FVa^307-506^ fragments in the human system. The rapid clearance of the FVa^1-506^ fragment and faster appearance of the smaller FVa^307-506^ fragment led us to speculate that their model rate constant for the cleavage at Arg^306^ was not appropriate for the human system of proteins. From a comparison of the empirical data to the initial *in silico* simulation we estimated that the rate of cleavage occurring at Arg^306^ was three times faster than the model predictions. To account for this the rate constant (Figure 2, k_4_; Table 1, Eqns 3 and 6) in the mathematical model was adjusted from 0.064 s^-1^ to 0.192 s^-1^ and simulations rerun for all analytes (Figure 3, solid lines). As expected the simulated predictions for FVa cofactor activity and FVa^HC^ were minimally altered by this change and continued to correlate well with their respective empirical measures (Figure 3, Panels A and C, solid lines vs. filled circles). The adjusted rate constant significantly altered the *in silico* predictions for both smaller FVa inactivation fragments (Figure 3, Panels D and E, solid lines vs. dashed lines) yielding a better fit with empirical results. The revised model using the adjusted kinetic rate was extended to experiments conducted with FVa_i_^506^ and the predicted time courses for the two models compared to the empirical results (Figure 3, Panel F, solid lines and dashed lines vs. open circles). As can be seen in all simulations, those using the published bovine model values (Figure 3, dashed lines) resulted in a poorer fit [33]. The rate constant for cleavage at Arg^306^, k_4_, was adjusted to 0.192 s^-1^ for all subsequent studies.

Our empirically driven adjustment to k_4_ results in a second order rate constant for the overall process of cleavage at Arg^306^ of 2.74 x 10^7^ M^-1^ s^-1^. Previously published second order rate constants for the APC cleavage at Arg^306^ in human FVa (1.55 x 10^6^ M^-1^ s^-1^[42], 1.7 x 10^6^ M^-1^ s^-1^[31], and 2.5 x 10^6^ M^-1^ s^-1^[41]) are an order of magnitude lower than our currently derived estimate and lower than that used by Hockin et al. (9.1 x 10^6^ M^-1^ s^-1^[33]) to describe the bovine system.

Studies using human proteins on APC inactivation of FVa reported second order rate constants for the cleavage at Arg^506^ ranging from 3.0-4.3 x 10^7^ M^-1^ s^-1^[31,41]; a more recent study by Nicolaes et al. [42] has reported the second order rate constant to be 1.17 x 10^8^ M^-1^ s^-1^. This value is quite similar to the value (1.42 x 10^8^ M^-1^ s^-1^) used in the current study.

An alternative approach to fitting these data sets would have been to use an optimization method in which all rate constants may be simultaneously adjusted to generate a best fit to all the experimental data. Failure of model predictions to fit the empirical data can stem from the collective effect of small errors in the rate constants used. It is not uncommon for laboratories to report 2–3 fold differences in rate constants of biochemical reactions and interactions measured *in vitro.* These ranges are accepted because the findings would fall within the margin of error expected for slightly variable experiments conducted in different laboratories, or for different techniques used to measure a rate constant. Such variation can have significant effects on model outputs [43]. However, lack of fit can arise from a fundamental mistake or omitted interaction in the reaction description. Therefore, we have not taken this approach as we developed this model.

### APC Inactivation of Factor Va: Effect of Factor Xa Under Non-saturating Conditions

It has been widely reported that FXa shields FVa from degradation by APC through its reversible association in the *prothrombinase* (FXaFVa) complex [34-38,44]. The mechanistic basis of this protection has been conceptualized as a mutually exclusive competition for association with membrane bound FVa, so that when FVa is associated with FXa it is not a substrate for APC [35], at least at the Arg^506^ site [37]. In order to develop a model description that can predict the extent of the protective effect exerted by FXa against APC proteolysis of FVa over a range of reactant concentrations, empirical experiments were performed at saturating and non-saturating concentrations of FXa.

#### Empirical Analyses

**Figure 4:**
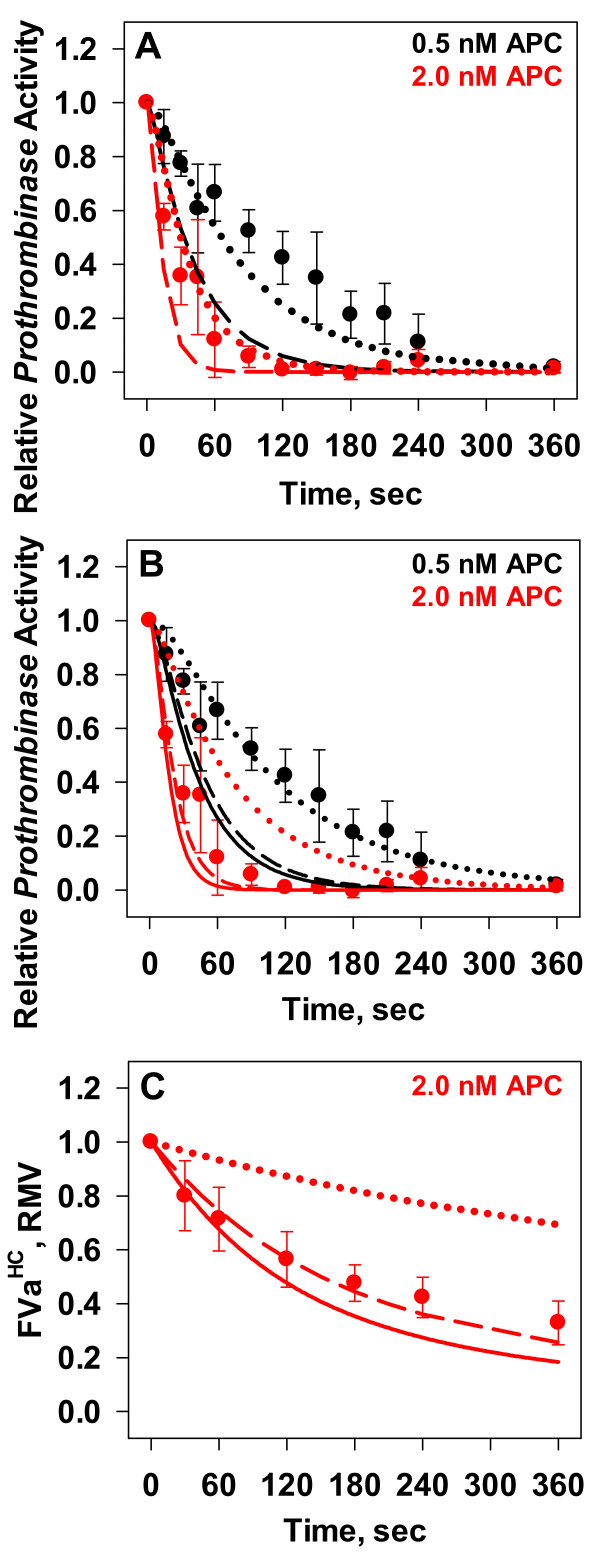
**Effect of Factor Xa on Activated Protein C Inactivation of Factor Va.** Empircal time courses of FVa inactivation measured by activity analysis (Panels *A* and *B*) or by monitoring proteolytic cleavages (Panel *C*) are presented along with corresponding simulated time courses. All data points are presented as averages ± S.D. of 4–6 experiments. (*A,B*) Empirical time courses of APC (0.5 nM – black circles; 2.0 nM – red circles) inactivation of preformed *prothrombinase* (200 pM FVa and 100 pM FXa). Corresponding simulated time courses for 0.5 nM (black) or 2.0 nM (red) APC are presented with three potential K_D_ values for the formation of the *prothrombinase* complex (100 pM – dotted lines; 500 pM – dashed lines; 750 pM – solid line): in Panel *A* each equilibrium process was described using a k_on_ (4.0 x 10^8^ M^-1^ s^-1^) and adjusting the k_off_ constant; in Panel *B* each equilibrium process was described using a k_on_ (1.5 x 10^8^ M^-1^ s^-1^) and adjusting the k_off_ constant. (*C*) Empirical time course of 2.0 nM APC inactivation of preformed *prothrombinase* (20 nM FVa and 30 nM FXa*) anaylzed by Western blotting. FVa^HC^ densitometric data are expressed as relative to its value at time 0 (red circles). Corresponding simulated time courses for 2.0 nM (red) APC are presented using three potential K_D_ values for the formation of the *prothrombinase* complex with each equilibrium process described by using a k_on_ (1.5 x 10^8^ M^-1^ s^-1^) and adjusting the k_off_ constant (100 pM – dotted lines; 500 pM – dashed lines; 750 pM – solid line).

In the first set of experiments, *prothrombinase* was preformed under non-saturating concentrations with FVa in excess (0.2 nM FVa and 0.1 nM FXa) on 20 μM PC:PS vesicles. Under these conditions, if the K_D_ = 0.5 nM for the (FXaFVa) complex (estimate based on previous studies [45]), only 13 % of the FVa would be expected to be bound to FXa. Inactivation reactions were initiated with 0, 0.5, or 2.0 nM APC. For these experiments, the amount of thrombin generated in the 0 nM APC control was set at 100 % *prothrombinase* activity, with thrombin generation levels represented relative to the *prothrombinase* activity in the 0 nM APC control. The loss of *prothrombinase* activity following the addition of APC at either concentration (Figure 4, Panel A, filled circles) appeared to follow a monophasic decay (fits not shown).

#### Mathematical Simulations

To simulate this experiment, six additional equations were required (Table 2, Eqns 11–16). Of these, four equations describe the formation of a *prothrombinase* complex between FXa and FVa (FXaFVa) or any of the three partially proteolyzed FVa species (FXaFVa_i_^506^, FXaFVa_i_^306^, and FXaFVa_i_^306/506^) using a K_D_ twice that describing the complex formed with intact FVa (FXaFVa) (based on previous studies [32]). Several empirical studies using plasma derived FVa and recombinant FVa mutants have demonstrated that FVa_i_^506^ and FVa_i_^306^ can complex with FXa and form a productive enzyme complex [31,32,41]. Two additional equations allow for the dissociation of heavy chain fragments from the *prothrombinase* species formed with either FVa_i_^306^ or FVa_i_^306/506^.

**Table 2:** Equations describing interactions between intact and partially proteolyzed factor Va with factor Xa and prothrombin

**Eqn**	**Chemical Expression**	**k**_**off**_	**k**_**on**_	**k**_**dis**_
		**(s**^**-1**^**)**	**(M**^**-1**^ **s**^**-1**^**)**	**(s**^**-1**^**)**
11	FXa + FVa ← 7-6 → FXaFVa	0.2 ^a^ 0.075 ^b^	4 x 10^8 a^	
			1.5 x 10^8 b^	
12	FXa + FVa_i_^506^ ← 8-6 → FXaFVa_i_^506^	0.4 ^c^	4 x 10^8 c^	
		0.15 ^b^	1.5 x 10^8 b^	
13	FXa + FVa_i_^306^ ← 8-6 → FXaFVa_i_^306^	0.4 ^c^	4 x 10^8 c^	
		0.15 ^b^	1.5 x 10^8 b^	
14	FXa + FVa_i_^306/506^ ← 8-6 → FXaFVa_i_^306/506^	0.4 ^c^	4 x 10^8 c^	
		0.15 ^b^	1.5 x 10^8 b^	
15	FXaFVa_i_^306^ -9 → FXa + FVa^1-306^FVa^LC^ + FVa^307-679/709^			0.0035
16	FXaFVa_i_^306/506^ -9 → FXa + FVa^1-306^FVa^LC^ + FVa^307-506^ + FVa^507-679/709^			0.0035
17	FVa + PT ← 11-10 → FVaPT	70^d^	1 x 10^8^	

^a^ Values from previously published studies for mathematical simulations [32,46].

^b^ Values identified in the current study for mathematical simulations and carried forward through all subsequent mathematical simulations.

^c^ Values are based on relative findings from previously published studies for mathematical simulations [32].

^d^ Initial estimate for FVaPT complex from Tran et al. [39].

Our initial mathematical simulations were carried out utilizing a K_D_ of 0.5 nM [45], resulting in an initial concentration of 26 pM for *prothrombinase*, with off and on rates of 0.2 s^-1^ and 4.0 x 10^8^ M^-1^ s^-1^, respectively (Table 2, Eqn 11) [46,47]. The mathematical predictions for the loss of *prothrombinase* activity in the presence of either 0.5 or 2.0 nM APC showed an approximately 2-fold faster loss in *prothrombinase* activity than observed in the empirical experiments (Figure 4, Panel A, dashed lines vs. filled circles).

To generate a better fit to the empirical data we first examined the dissociation constant of FXa and FVa on PC:PS vesicles, which by literature reports varies from as low as 83 pM [31] to as high as 1 nM [48]. Simulations to test the wide range of reported K_D_ values were first conducted by altering the k_off_ rate constants for the *prothrombinase* complex, while maintaining the same k_on_ rate constant of 4.0 x 10^8^ M^-1^ s^-1^. For each K_D_ value tested, the starting concentrations of free and bound species (FVa, FXa, and FXaFVa) were recalculated and used as the initial conditions for a mathematical simulation. An adjustment made to the off rate for the *prothrombinase* complex resulting in a K_D_ of 0.1 nM (58.8 pM *prothrombinase* initially) generated an *in silico* prediction that more closely represented the empirical results seen with both 0.5 and 2.0 nM APC (Figure 4, Panel A, black and red, respectively, dotted lines vs filled circles).

In addition to altering the k_off_ rate constant while maintaining a k_on_ rate constant to reach a desired overall K_D_, changes to both isolated rate constants were explored as a potential solution. One alternative that we explored was adjusting the k_on_ rate constant to 1.5 x 10^8^ M^-1^ s^-1^ and setting the k_off_ rate constant to values that yielded the same set of K_D_ values. Figure 4, Panel B presents these model results at 0.5 and 2.0 nM APC (black and red, respectively) for K_D_'s of 0.5 nM (dashed lines) and 0.1 nM (dotted lines) for *prothrombinase* assembly. In contrast to simulations based with a k_on_ value of 4.0 x 10^8^ M^-1^ s^-1^ this analysis indicates that a K_D_ of 0.5 nM yields a better fit to the data.

These results highlight an important feature of dynamic systems like this one: the magnitude of the observed protective effect may depend not only on the concentrations of the three protein components and the K_D_'s characterizing their interactions, but also on the rate constants defining the competing K_D_'s.

### APC Inactivation of Factor Va: Effect of Factor Xa Under Saturating Conditions

**Figure 5:**
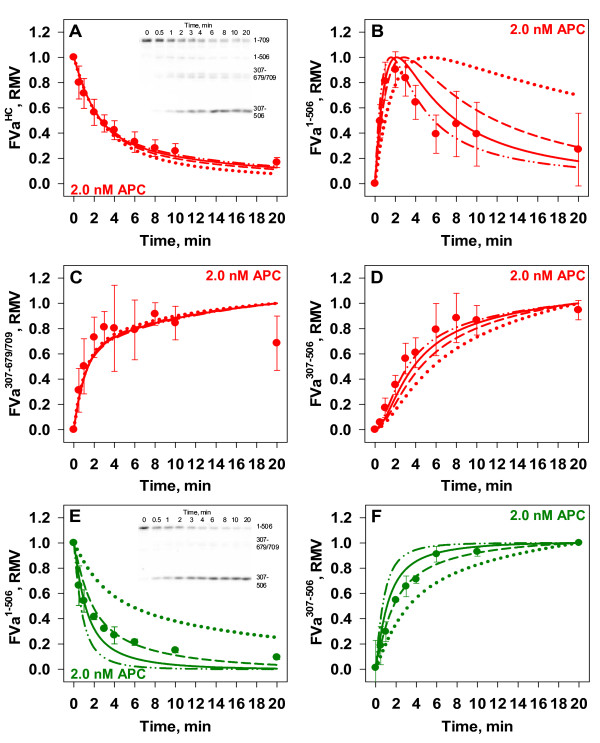
**APC Inactivation of FVa in the Presence of a Saturating Concentration of FXa.** Empirical: FVa (20 nM, Panels *A-D*) or FVa_i_^506^ (20 nM, Panel *E* and *F*) was pre-incubated with active site blocked FXa* (30 nM) at 37°C. The reaction was then initiated with 2.0 nM APC. The inactivation of FVa was monitored by densitometry for the following fragments: FVa^HC^ (Panel *A*), FVa^1-506^ (*B*), FVa^307-679/709^ (*C*) and FVa^307-506^ (*D*) and the inactivation of FVa_i_^506^ was monitored by following FVa^1-506^ (*E*) and FVa^307-506^ (*F*). All data points with FVa are presented as averages ± S.D. of 4–6 experiments and data points with FVa_i_^506^ are presented as averages ± S.D. of 2 experiments. Mathematical: Simulated time courses in which the model description does not allow APC proteolysis of the FXaFVa_i_^506^ complex (dotted line) or allows the cleavage by APC at Arg^306^ in the FXaFVa_i_^506^ complex with rate constants of 2.5 x 10^6^ M^-1^ s^-1^ (dashed line), 5.0 x 10^6^ M^-1^ s^-1^ (solid line), or 9.1 x 10^6^ M^-1^ s^-1^ (dash with dotted line).

The previous analysis under non-saturating conditions indicates that either a dissociation constant of 0.1 nM or 0.5 nM for the *prothrombinase* complex can recapitulate our empirical data depending on the values assigned to k_on_ and k_off_. From the point of view of empirically measuring k_on_ and k_off_ values the differences in these assigned values (e.g. ~2.5 fold between 1.5 or 4.0 x 10^8^ M^-1^ s^-1^) are small and within the margin of error for this type of analysis. In order to further explore which dissociation constant and set of rate constants is appropriate for the empirical data, we tested APC inactivation of FVa under saturating conditions with FXa present in excess (Figure 4, Panel C and Figure 5).

#### Empirical Analyses

Figure 5 presents empirical experiments in which preformed *prothrombinase* (20 nM FVa and 30 nM active site blocked FXa (FXa*)) was treated with 2.0 nM APC and time courses of FVa^HC^ and its APC-derived fragments FVa^1-506^, FVa^307-679/709^ and FVa^307-506^ visualized by Western blotting (Figure 5, Panel A, inset). Under these conditions, 95 % to 99 % of the FVa is bound to FXa* prior to addition of APC given K_D_ values in the range of 0.5 nM to 0.1 nM, respectively. The selection of 20 nM FVa reflected the fact that in closed model systems of TF-initiated coagulation, levels of FVa reach 20 nM [49] and the practical requirement for a concentration suitable for Western blot analysis. Active site blocked FXa was used to prevent FXa proteolysis of FVa [50].

Reactions where the FVa population is highly associated with FXa* showed 70 % of FVa^HC^ remained after 1 minute (Figure 4, Panel C and Figure 5, Panel A, red circles) compared to ~ 10 % without FXa* (Figure 3, Panel C, red circles). Comparison of initial rates showed a greater than 7-fold reduction in the initial rate of 2.0 nM APC proteolysis of FVa due to its FXa association (77 pM/s vs. 558 pM/s). Consistent with this overall suppression of heavy chain proteolysis, generation of the FVa^1-506^ fragment was slower in the presence of FXa*, reaching a maximum in 2 min vs ~30 s in its absence (Figure 5, Panel B vs. Figure 3, Panel D, red circles). Its clearance was also suppressed, taking ~ 4 min to decline to 50 % of its maximum as opposed to ~1 min without FXa* (Figure 5, Panel B vs. Figure 3, Panel D, red circles).

In contrast to APC inactivation studies conducted in the absence of FXa*, visual inspection of Western blot images (Figure 5, Panel A, inset) reveal the accumulation of the FVa^307-679/709^ fragment leading to more reliable quantitative densitometric analysis of this fragment (Figure 5, Panel C, red circles). Generation of this fragment reaches 50 % of its maximal level by 1 minute, and remains at elevated levels above 70 % of maximal level after 2 minutes (Figure 5, Panel C, red circles). Generation of the FVa^307-506^ fragment reaches 50 % of maximal value after 3 minutes (Figure 5, Panel D, red circles).

#### Mathematical Simulations

Initial *prothrombinase* concentrations given the condition of pre-incubating 20 nM FVa and 30 nM FXa* were calculated to be 19.1 nM or 19.8 nM using K_D_ values of either 0.5 nM or 0.1 nM, respectively. A range of k_on_ and k_off_ rate constants for each K_D_ were tested by varying k_on_ values between 1 and 4.0 x 10^8^ M^-1^ s^-1^. Time courses of the inactivation of FVa by 2.0 nM APC were produced. The results of this *in silico* study indicated that the disappearance of FVa^HC^ was dependent on the resultant K_D_ value and independent of the combination of k_on_ and k_off_ rate constants used to generate the K_D_ values of 0.1 or 0.5 nM (data not shown). Assuming the K_D_ for *prothrombinase* is 0.1 nM and the k_on_ is either 1.5 x 10^8^ M^-1^ s^-1^ or 4.0 x 10^8^ M^-1^ s^-1^, the model predicts that after 6 min 25 % of the FVa^HC^ is proteolyzed (Figure 4, Panel C, red dotted line), in contrast to the empirical results showing ~82 % of FVa^HC^ proteolyzed at 6 minutes (Figure 4, Panel C, red circles). Model simulations with K_D_ values of 0.5 nM, 0.75 nM or 1.0 nM resulted in FVa^HC^ levels decreasing after 6 min by 75 % (Figure 4, Panel C, red dashed line), 83 % (Figure 4, Panel C, red solid line), or 87 % (data not shown), respectively. From the mathematical simulations, the initial rates of FVa^HC^ proteolysis were 22 pM/s, 86 pM/s, and 122 pM/s for K_D_’s of 0.1 nM, 0.5 nM, and 0.75 nM, respectively, compared to the empirically derived rate of FVa^HC^ proteolysis of 78 pM/s. Overall these comparisons support our eliminating 0.1 nM as the K_D_ for the *prothrombinase* complex.

While the mathematical model is able to recapitulate the overall combined initial cleavages of FVa^HC^ (Figure 5, Panel A, dotted line vs. filled circles), there is a mixed success for the predictions of the inactivation fragments (Figure 5, Panels B-D, dotted line vs. filled circles). Specifically, the mathematical model captures the rapid accumulation of the FVa^307-679/709^ fragment (Figure 5, Panel C, dotted line), but does not capture the clearance of the FVa^1-506^ fragment (Figure 5, Panel B, dotted line) or the generation of the FVa^307-506^ fragment (Figure 5, Panel D). The same lack of fit for the proteolysis of this fragment is observed when FVa_i_^506^ is the starting substrate (Figure 5, Panels E and F, dotted line vs. filled circles).

In the mathematical model as constructed APC proteolysis at any site is permitted only with FVa species not bound to FXa (or FXa*). The more rapid clearance of FVa^1-506^ observed empirically suggests two possible problems with the model construct: 1) the binding affinity between FVa_i_^506^ and FXa is weaker than the model value of 1 nM based on studies with bovine FVa [32] or 2) APC can cleave at Arg^306^ when the FVa_i_^506^ species is bound to FXa. Previous studies with recombinant human proteins have reported K_D_ values ranging from ~3.9 nM [31] to ~1.35 nM [51]. To evaluate the likelihood that a weaker binding affinity was the culprit, mathematical simulations were conducted where the K_D_ for the FXaFVa_i_^506^ complex was varied; to fit the empirical data the K_D_ for this complex would have to be greater than 10 nM (data not shown) and as such this adjustment was discarded as a viable option.

The alternative explanation for the empirical data presenting the clearance of the FVa^1-506^ fragment is that when bound to FXa, the FVa_i_^506^ species is susceptible to APC cleavage at Arg^306^. To explore this hypothesis, an additional reaction was added to the mathematical construct: APC cleaving (FXaFVa_i_^506^) at Arg^306^. To establish whether there was a value for this rate constant that would improve the fit, simulations varying its value were run using the model value (2.7 X 10^7^ M^-1^ s^-1^) for the cleavage of Arg^306^ in free FVa as a point of reference. Representative examples are presented in Figure 5. The mathematical analysis suggests that if the Arg^306^ in (FXaFVa_i_^506^) is a target for APC, the magnitude of the rate constant regulating the event is ~10 to 20 % that for free FVa.

#### Structural Analysis

**Figure 6:**
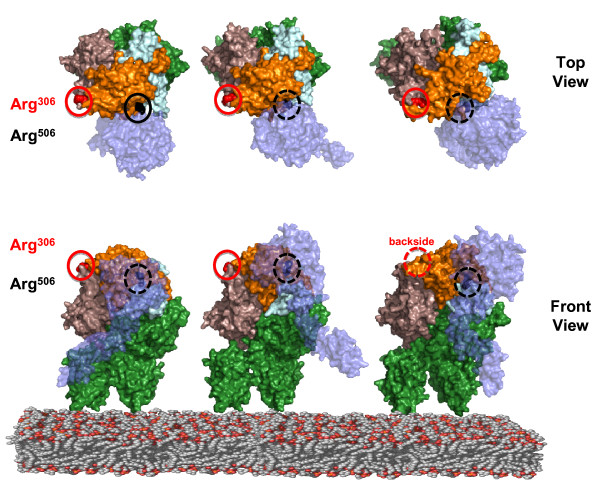
**Structural Simulations of the*Prothrombinase*Complex.** Top and front views of the structural simulations of the *prothrombinase* complex from three separate groups: *Left,*[52], *Center*[53], *Right*[54]. In the three models the FXa structures (presented in light blue) are based on previously solved crystal structures [PDB ID: 1XKA] [55]. In all three simulations, the FVa domain orientation is based on the crystal structure of bovine inactivated FVa_i_ (FVa^1-306^FVa^LC^) solved by Adams et al. [PDB ID: 1SDD] [56]. The FVa^1-306^ fragment is presented in purple, the FVa^LC^ is presented in green, and the hypothetical structure of FVa^307-709^, which is based on the homology comparisons to the structure of human ceruloplasmin [PDB ID: 1KCW] [57], is presented in orange. The Arg^306^ residue is marked in red and encircled by a red circle and with the Arg^506^ residue in black and encircled by a black circle; dashed circles indicate the residue in question is completely masked by a structure from the presented view point.

Analysis of the proposed structural models of the *prothrombinase* complex allows for the possibility that Arg^306^ is susceptible to APC in the FXaFVa_i_^506^ complex [52-54]. The *prothrombinase* complex models differ in the orientation of FXa relative to FVa, and the binding interface between enzyme and cofactor. What is consistent between the three models is that Arg^506^ in FVa is occluded by the binding of FXa, rendering this site inaccessible to APC cleavage (Figure 6). Additionally Arg^306^ is surface exposed and not masked by FXa in any of the models. As such, the structural models suggest that this arginine site could be accessible to APC cleavage even when FVa or FVa_i_ species are complexed to FXa. However, mathematical simulations allowing the cleavage to occur in FVa while bound to FXa overestimate both the rate of FVa^HC^ disappearance and the rate of generation of FVa^307-679/709^ fragment observed empirically (data not shown). The data was best fit by modeling only the FXaFVa_i_^506^ complex as susceptible to cleavage at Arg^306^. Thus the kinetic and structural modeling in concert with the empirical data suggests two effects: binding of FVa to FXa blocks both cleavage at Arg^306^ and Arg^506^ despite the unmasked surface exposure of Arg^306^; and that binding of FXa to FVa_i_^506^ improves the geometry at Arg^306^ rendering the site vulnerable to APC.

### APC Inactivation of Factor Va: Effect of Prothrombin

In addition to the protection afforded to FVa by FXa in the *prothrombinase* complex, the substrate of the complex, prothrombin (PT), has also been shown to inhibit APC inactivation of FVa [39,40,58]. Guinto and Esmon reported that prothrombin protects the intact FVa molecule by competing with APC for binding to FVa [58]; more recent studies have reported a K_D_ of between 500–700 nM for FVa and prothrombin [39,40].

#### Empirical Analyses

**Figure 7:**
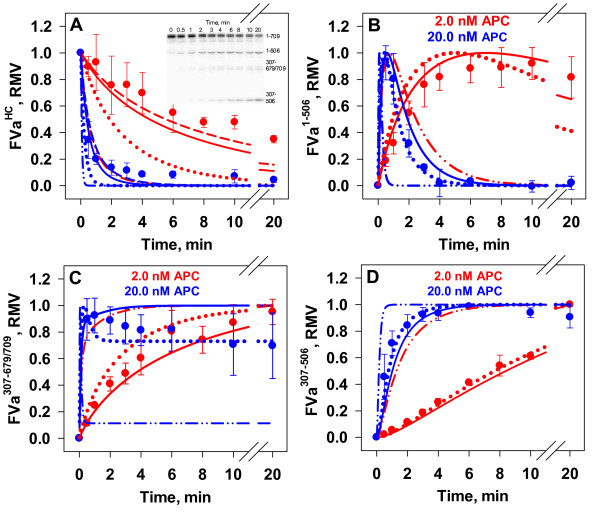
**Effect of Prothrombin on the APC Inactivation of FVa.** Empirical: FVa (20 nM) was pre-incubated with prothrombin (1.4 μM) at 37°C. The reaction was initiated with 2.0 nM (red circles) or 20.0 nM (blue circles) APC. A representative Western blot is shown in the Panel *A* inset. Data are normalized densitometric values for the following fragments: FVa^HC^ (Panel *A*), FVa^1-506^ (*B*), FVa^307-679/709^ (*C*) and FVa^307-506^ (*D*). Data points are the averages ± S.D. of either 4 or 3 experiments for inactivation studies conducted with either 2.0 or 20.0 nM APC, respectively. Mathematical: Simulated time courses (2.0 nM APC – red lines; 20.0 nM APC – blue lines) were generated under two modeling conditions: 1) The assumption of unlimited binding sites for all species and a K_D_ of 700 nM for FVaPT complex (dash and dotted lines) [39]; and 2) The assumption of limited binding sites, thus reducing the effective APC concentration, with hypothetical K_D_ values for the FVaPT complex of 0 nM (dotted line), 500 nM (dashed line), or 700 nM (solid line) are shown.

In order to quantify the degree to which prothrombin protects FVa from APC, reactions were carried out at physiological concentrations of FVa (20 nM) and prothrombin (1.4 μM) on phospholipid vesicles (20 μM). Reactions were triggered with APC (2.0 or 20.0 nM) and aliquots removed for Western blot analysis (representative blot in inset of Figure 7, Panel A). The presence of prothrombin in the system clearly protected FVa from APC inactivation by 2.0 nM APC. After 20 minutes 35 % of FV^HC^ remained intact (Figure 7, Panel A, red circles), compared to less than 5 % intact within 6 minutes in the absence of prothrombin (Figure 3, Panel C, red circles). The initial rate of proteolysis of the heavy chain was reduced ~ 16-fold (34 pM/s vs. 558 pM/s). The formation of the FVa^1-506^ fragment (Figure 7, Panel B) and rate of FVa^307-506^ fragment (Figure 7, Panel D) were both suppressed relative to reactions without prothrombin (Figure 3 Panels D and E, respectively) suggesting both sites were protected. At 20 nM APC (Figure 7, Panel A, blue circles) the initial rate of FVa^HC^ proteolysis (520 pM/s) is similar to that observed with 2.0 nM APC in the absence of prothrombin (558 pM/s). Contrary to inactivation experiments in the absence of prothrombin (Figure 3, Panel B), there is a more noticeable generation of the FVa^307-679/709^ fragment (Figure 7, Panel A inset) that enables more reliable densitometric quantitation (Figure 7, Panel C).

#### Mathematical Simulations

An additional reaction describing the reversible formation of the FVaPT complex with a K_D_ of 700 nM [39] was added to the ordinary differential equation (ODE) network (Table 2, Eqn 17). The initial concentration of preformed FVaPT complex, given 20 nM FVa and 1.4 μM PT, was calculated to be 13.3 nM. Simulated time courses for FVa heavy chain fragments generated by APC (2.0 nM or 20.0 nM, red and blue lines, respectively) proteolysis did not correlate well with the empirical data, in fact suggesting that there would be little to no protection of FVa by 1.4 μM prothrombin if the K_D_ was 700 nM (Figure 7, Panel A, dashed with dotted lines vs. filled circles; Note, red dashed with dotted line is underneath blue dashed line). Test simulations varying the K_D_ were performed by altering the k_off_ constant for the equilibrium describing the prothrombin and FVa interaction. Even when a K_D_ of 50 nM was assigned to the interaction, ~ 10-fold less than published estimates [40] the resulting simulations did not capture the observed level of protection (data not shown).

In the computational model one of the assumptions is the presence of unlimited binding sites located on a single surface so that competition between proteins for occupancy of the phospholipid surface is not a factor. At 20 μM PC:PS vesicles, and setting the phospholipid/protein binding site ratio between 30 and 60 [59-61], available binding sites fall in a range between 220 and 440 nM. The simultaneous solution of the equilibrium expressions for prothrombin, FVa, and APC (defined as non-catalytic) binding to phospholipid membrane with K_D_’s of 230 nM [62], 2.72 nM [63], and 500 nM [64], respectively, shows that between 77–78 % of binding sites are occupied by prothrombin, over 96 % of the FVa is bound, and only approximately 11 % of the APC is able to bind to the surface whether 2.0 or 20.0 nM APC is present. By using this bound fraction of APC as the catalytically relevant population, the mathematical simulations better captured the kinetics of FV^HC^ proteolysis in the presence of 1.4 μM prothrombin (Figure 7, Panel A, solid lines vs. filled circles). Similarly, comparison of empirical and simulated time courses for the three generated fragments (Figure 7, Panels B-D, filled circles vs. solid lines) also show improved fits. Our findings indicate that there may in fact be a tighter association between FVa and PT, in line with the findings of Yegneswaran et al. (Figure 7. Panel A, filled circles vs. dashed lines) [40].

To verify that the impaired FVa proteolysis observed under the empirical reaction conditions was not solely due to competition for phospholipid binding sites, mathematical simulations were constructed using the adjusted APC concentration, but without permitting the formation of the FVaPT complex. This model construct failed to predict the time courses for FVa^HC^ disappearance and the fragments FVa^1-506^ and FVa^307-679/709^ (Figure 7, Panel A, B and C, filled circles vs. dotted lines). Thus the computational studies indicate that the prothrombin dependent suppression of FVa proteolysis by APC observed in our empirical reactions is due both to binding site competition (prothrombin membrane association limiting APC access to the membrane surface) and to the FVaPT complex not being a substrate for APC. Comparisons of the empirical data for FVa^HC^ proteolysis (Figure 7, Panel A) with the simulations representing unlimited binding sites (dashed with dotted lines), limited binding site adjustment but no FVaPT interaction (dotted line) and the limited binding site adjustment with FVaPT complex formation (K_D_ = 700 nM) (solid line) indicate that under these conditions approximately half of the observed PT dependent suppression is due to FVaPT complex formation.

### APC Inactivation of Factor Va: Effect of Prothrombin and Saturating Levels of Factor Xa

#### Empirical Analysis

**Figure 8:**
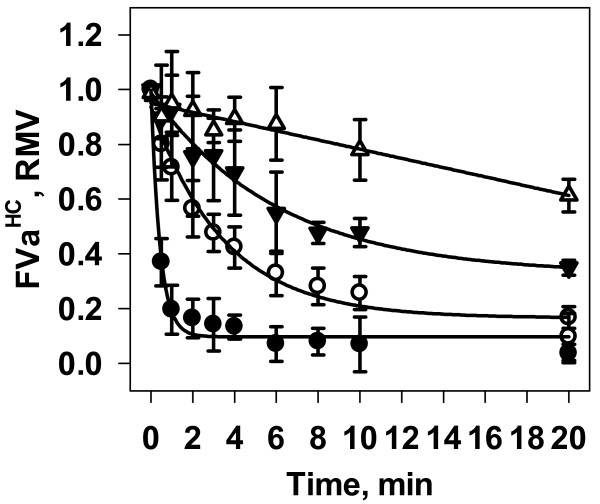
**Effect of*Prothrombinase*Components on APC Proteolysis of FVa.** Normalized densitometry data measuring the disappearance of the FVa^HC^ are from reactions containing 20 μM PC:PS, 2.0 nM APC and 20 nM FVa (filled circles, N = 6), with either 30 nM active site blocked FXa (open circles, N = 5), 1.4 μM prothrombin (filled triangles, N = 4), or 30 nM active site blocked FXa + 1.4 μM prothrombin (open triangles, N = 4). Solid lines are fits of the empirical data to monophasic exponential decay curves (FVa, FVa + FXa*, and FVa + PT) or a linear decay (FVa + FXa* + PT).

In order to measure the cumulative protection prothrombin and saturating levels of active site blocked FXa have on the APC inactivation of FVa, reactions were carried out at physiological concentrations of FVa (20 nM) and prothrombin (1.4 μM), with saturating levels of FXa* (30 nM) on phospholipid vesicles (20 μM). Figure 8 summarizes the time course data for FVa^HC^ proteolysis by 2.0 nM APC for reactions constructed with FVa, FVa + FXa*, FVa + PT, and FVa + FXa* + PT. The combined presence of prothrombin and FXa* provided maximum protection against APC inactivation.

**Figure 9:**
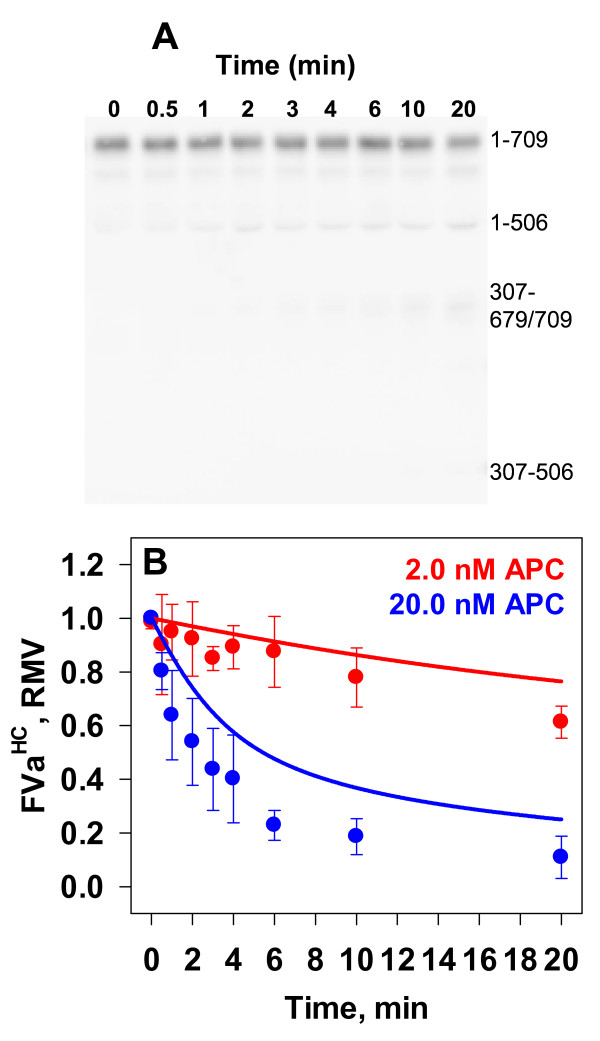
**APC Inactivation of FVa in the Presence of Prothrombin and Saturating Levels of FXa.** Empirical: FVa (20 nM) was pre-incubated with 30 nM active site blocked FXa (FXa*) and prothrombin (1.4 μM) at 37°C. The reaction was initiated with 2.0 nM (red circles) or 20.0 nM (blue circles) APC. A representative Western blot from an empirical experiment using 2.0 nM APC is shown in Panel *A*. Densitometric analysis of normalized measurements of the FVa^HC^ are presented in Panel *B*. Data points are the averages ± S.D. of either 4 or 3 experiments for inactivation studies conducted with either 2.0 or 20.0 nM APC, respectively. Mathematical: Simulated time courses (2.0 nM APC – red lines; 20.0 nM APC – blue lines) were generated with the assumption of limited binding sites, resulting in an effective APC concentration of 11 % (solid line) is shown.

Figure 9 presents the empirical data comparing the proteolysis of FVa in the presence of 30 nM FXa* and 1.4 μM PT at 2.0 nM and 20.0 nM APC. Figure 9, Panel A shows a representative Western blot for the reactions with 2.0 nM APC. Visual inspection of Western blots revealed extremely low generation of the inactivation fragments FVa^1-506^, FVa^307-679/709^, and FVa^307-506^. Productions of these fragments were consistently barely above detection limits and thus made measurement of these fragments highly variable, and as a result were not considered further.

Addition of 2.0 nM APC to the reaction system led to only 39 % of the FVa^HC^ being proteolyzed after 20 minutes (Figure 9, Panel B, red circles). The initial rate of disappearance was 14 pM/s, nearly 41-fold lower than the inactivation rate in the absence of both prothrombin and FXa (558 pM/s; Figure 3, Panel A, red circles). Inactivation studies with 10 times more APC resulted in a nearly 8-fold increase in the initial rate of inactivation (100 pM/s) (Figure 9, Panel B, blue circles).

#### Mathematical Simulations

To incorporate the formation of the ternary *prothrombinase*substrate complex, four additional reactions are required (Table 3, Eqns 19–22). Based on studies indicating that *prothrombinase* complexes formed with partially proteolyzed FVa species have a K_M_ similar to the complex formed with intact FVa [31,51], we set the K_M_ of prothrombin for any *prothrombinase* complex to be the same and consistent with previous modeling studies [46,47]. We included two additional reactions (Table 3, Eqns 23–24) to allow for the dissociation of the ternary complex formed with some of the partially proteolyzed FVa species. Solving the simultaneous equilibrium of FVa, FXa*, and PT in a lipid independent system, approximately 18.9 nM FVa is found in a *prothrombinase* complex (FVaFXa or FVaFXaPT), approximately 720 pM FVa is in the FVaPT complex, and approximately 360 pM FVa is unassociated.

**Table 3:** **Equations describing interactions between catalytically inactive*prothrombinase*species and prothrombin**

**Eqn**	**Chemical Expression**	**k**_**off**_	**k**_**on**_	**k**_**dis**_
		**(s**^**-1**^**)**	**(M**^**-1**^ **s**^**-1**^**)**	**(s**^**-1**^**)**
19	FXaFVa + PT ← 14-13 → FXaFVaPT	103 ^a^	1.0 x 10^8 a^	
20	FXaFVa_i_^506^ + PT ← 14-13 → FXaFVa_i_^506^PT	103	1.0 x 10^8^	
21	FXaFVa_i_^306^ + PT ← 14-13 → FXaFVa_i_^306^PT	103	1.0 x 10^8^	
22	FXaFVa_i_^306/506^ + PT ← 14-13 → FXaFVa_i_^306/506^PT	103	1.0 x 10^8^	
23	FXaFVa_i_^306^PT −9 → FXa + FVa^1-306^FVa^LC^ + FVa^307-679/709^ + PT			0.0035
24	FXaFVa_i_^306/506^PT −9 → FXa + PT + FVa^1-306^FVa^LC^ + FVa^307-506^ + FVa^507-679/709^			0.0035

^a^ Values from previously published studies for mathematical simulations [32,46].

Simulated time courses for the disappearance of the FVa^HC^ were generated for the experiments where 2.0 nM or 20.0 nM APC was added. Based on the findings from the APC inactivation studies of FVa in the presence of prothrombin, APC effective concentrations were adjusted to 11 % of the total to account for the limited binding sites (Figure 9, Panel B, solid lines). The simulations’ initial rates of proteolysis of the FVa^HC^ (6 pM/s) were less than half the measured empirical rates (14 pM/s). The mathematical simulations greater level of protection extends through 20 minutes with higher amounts of FVa^HC^ anticipated compared to empirical results (2.0 nM APC: 76 % vs. 61 %, respectively; 20.0 nM APC: 26 % vs. 11 %, respectively) (Figure 9, Panel B). Empirical studies were also conducted with 50 μM PC:PS vesicles and did not result in any significant changes in the initial rate or overall time course of FVa^HC^ proteolysis by 2.0 nM APC (data not shown), suggesting that under the current conditions, phospholipid membrane accessibility is not impeding the inactivation reaction.

Potential explanations for this disparity include: 1) accumulated effect of small errors in rate constants; 2) improved binding of APC to the surface; 3) a misestimate of the affinity of the *prothrombinase*prothrombin interaction; and 4) the affinity of human APC for FVa is slightly higher, e.g. the K_D_ is less than 7 nM. Additional experiments will be required to distinguish between these possibilities. It is important to note that phospholipid composition plays an important role in protein-membrane interactions which can extend to effects on enzyme activity (Reviewed in [65]). Several studies have directly highlighted the effect of membrane composition on the APC inactivation of FVa [66-68].

## Conclusions

**Figure 10:**
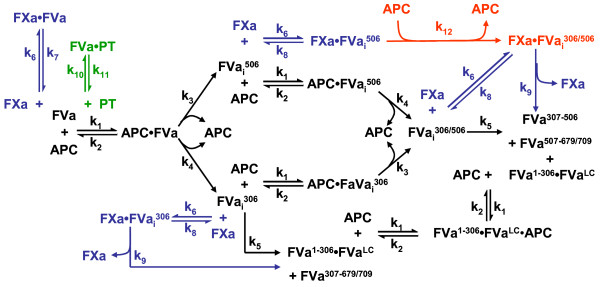
**Reaction Scheme of APC Inactivation of FVa in the Presence of Either FXa or Prothrombin.** Schematic of reactions and interactions involved in the APC inactivation of FVa when in the presence of either FXa (highlighted in blue and red) or prothrombin (highlighted in green). The interaction highlighted in red was identified in this study through the validation of the mathematical model describing the APC inactivation of FVa. Formation of the *prothrombinase* plus prothrombin species (Table 3, Eqns 19–22) and their dissociation (Table 3, Eqns 23–24) are not shown in this figure for clarity.

We have constructed an ODE based model of APC inactivation of the *prothrombinase* complex. This model includes 24 chemical reactions and interactions with 14 unique rate constants which describe the flux in concentrations of 24 species (Figure 10). We did so in stepwise fashion, analyzing the time course of FVa inactivation in empirical reaction systems with increasing number of interacting components and generating corresponding model constructs of each reaction system. Reaction mechanisms, rate constants and equilibrium constants informing these model constructs were initially derived from various research groups reporting on APC inactivation of FVa in isolation [22,31-33], in the presence of FXa [14,34-38], and in the presence of prothrombin [39,40,58]. Model predictions were assessed against empirical time course data measuring the appearance and disappearance of multiple FVa degradation intermediates as well as *prothrombinase* activity changes, with experiments done multiple times, with plasma proteins derived from multiple preparations. Current coagulation models that incorporate the protein C pathway [69-79] provide an often simplistic one step inactivation reaction of FVa by APC, without incorporating the feedback inhibition of the APCFVa^1-306^FVa^LC^ complex, the ability of partially proteolyzed FVa species to form catalytically active *prothrombinase* species, and the formation of FVaPT species. They either do not provide empirical data verifying their model construct or rely on a single global output to validate the entire model construct. Wagenvoord et al. have outlined the limitations of mathematical models of complex reaction networks validated by a single analyte [80]. This study highlights the complexity of the inactivation process and is a crucial step towards creating a module of equations describing the PC pathway that can be integrated into existing comprehensive mathematical models describing the interplay between procoagulant and anticoagulant processes during tissue factor initiated coagulation.

## Methods

### Reagents and Proteins

Human prothrombin and Factor X were isolated according to methods as described [81]. Factor Xa was prepared as previously described using Russel’s Viper Venom Factor Xa Activator [82,83]. Human Factor V was purified from citrated plasma according to previously described procedures [83]. Activated human protein C was purchased from Haematologic Technologies, Essex Junction, VT. 1,2-Dioleolyl-sn-Glycero-3-Phospho-L-Serine (PS) and 1,2-Dioleoyl-sn-Glycero-3-Phosphocholine (PC) were purchased from Avanti Polar Lipids, Inc (Alabaster, AL) and phospholipid vesicles (PC:PS) composed of 75 % PC and 25 % PS were prepared as described [60,84]. Spectrozyme TH and recombinant hirudin were purchased from American Diagnostica, Inc (Greenwich, CT) and EDTA was purchased from Sigma (St Louis, MO). D-Phe-Pro-ArgCH_2_Cl (FPRck) was prepared in house. Monoclonal anti-fV (αHFV#17) was obtained from the Biochemistry Antibody Core Laboratory (University of Vermont) and a goat anti-mouse IgG conjugated to HRP was purchased from Southern Biotech (Birmingham, AL). Active site blocked FXa (FXa*) was produced according to the previously published method [85].

Multiple preparations of FV/FVa and different lots of APC were utilized for these studies. Preparations of Factor Va were made fresh prior to all experiments. Factor V (1.0 μM) in 0.2 M HEPES, 0.15 M NaCl, 0.1 % PEG-8000, 2.0 mM CaCl_2_, pH 7.4 (HBS-PEG-Ca) was activated with 10 nM human thrombin for 10 min at 37°C. Thrombin activation was stopped by addition of 12 nM recombinant hirudin and placing the sample on ice. Factor Va preparations were used within 4 hours. The partially proteolyzed species of factor Va cleaved only at Arg^506^ was generated by incubating FVa (750 nM) in HBS-PEG-Ca with 5.0 nM APC for 20 minutes at 37°C, after 20 minutes an additional 5.0 nM APC was added. Following the inactivation reaction, the FVa_i_^506^ containing reaction was placed on ice and incubated with 1.0 mM di-isopropyl phosphate to inactivate the APC.

### Inactivation of Factor Va by Activated Protein C

Solutions of factor Va (20 nM) in HBS-PEG-Ca with 20 μM PC:PS were prepared at 37°C. At the zero time point 0.5 or 2.0 nM APC was added to the FVa solution. At designated intervals samples were removed, and either quenched in denaturing sample preparation buffer (0.31 M Tris[hydroxymethyl]aminomethane, 10 % sodium dodecyl sulfate, 50 % glycerol, 0.5 % Bromophenol Blue, pH 6.8) and analyzed either for proteolytic cleavage using SDS-PAGE and subsequent immunoblot analysis (Western blotting), or for co-factor activity using a one-stage clotting assay in factor V deficient plasma. Inactivation reactions were diluted 40-fold in an appropriate volume of factor V deficient plasma and assayed immediately. Clotting activity is represented as a percentage of the clotting time observed for unproteolyzed FVa.

### Inactivation of Factor Va by Activated Protein C in the Presence of Factor Xa

#### Activity analysis

Rates of APC inactivation of FVa (0.2 nM) in the presence of PC:PS vesicles (20 μM) and FXa (0.1 nM) were assessed with the *prothrombinase* complex preformed for five minutes at 37°C in HBS-PEG-Ca. At the zero time point APC (0–2.0 nM) was added to the *prothrombinase* complex solution. At designated intervals aliquots were removed and added to a minimal amount of concentrated prothrombin (1.0 μM final). After 90 s, aliquots were quenched with the addition of 0.5 M EDTA (25 mM final) and subsequently monitored for thrombin activity against the chromogenic substrate SpecTH. Thrombin concentrations were determined by reference to a standard curve and the level of thrombin generated used as a relative measurement of *prothrombinase* concentration compared to measurements with intact FVa.

#### Proteolytic analysis

*Prothrombinase* complex was preformed on 20 μM PC:PS vesicles in HBS-PEG-Ca for four minutes at 37°C using 30 nM fluorescein-active site blocked FXa (FXa*) and 20 nM FVa. A sample aliquot was removed, APC added (2.0 nM), aliquots taken at selected time points and quenched into denaturing sample preparation buffer, and subsequently analyzed by Western blotting.

### Inactivation of Factor Va by Activated Protein C in the Presence of Prothrombin

Reaction mixtures of FVa (20 nM) and prothrombin (1.4 μM) were prepared with 20 μM PC:PS vesicles in HBS-PEG-Ca and incubated for four minutes at 37°C. A sample aliquot was removed, APC added (2.0 or 20.0 nM), aliquots taken at selected time points and quenched into denaturing sample preparation buffer, and subsequently analyzed by Western blotting.

### Inactivation of Factor Va by Activated Protein C in the Presence of Prothrombin and Factor Xa

Reaction mixtures of FVa (20 nM), fluorescein-active site blocked FXa (FXa*, 30 nM), and prothrombin (1.4 μM) were prepared with 20 μM PC:PS vesicles in HBS-PEG-Ca and incubated for four minutes at 37°C. A sample aliquot was removed, APC added (2.0 or 20.0 nM), aliquots taken at selected time points and quenched into denaturing sample preparation buffer, and subsequently analyzed by Western blotting.

### Western blotting and densitometric analyses

All FVa samples were run under reducing conditions. FVa heavy chain and inactivation fragments were analyzed by fractionation on 4-12 % SDS-PAGE acrylamide slab gels followed by electrophoretic transfer of proteins to Immobilon-FL membranes (Millipore Inc., Billerica, MA), and detected using a mouse monoclonal antibody (αHFV#17) that binds to an epitope between residues 307–506 of the factor Va heavy chain and a goat anti-mouse IgG conjugated to HRP (Southern Biotech). Luminescence was detected using a Fuji LAS-4000 in conjunction with ImageCapture software (Fujifilm, Tokyo, Japan). Densitometry was carried out using MultiGauge software (Fujifilm, Tokyo, Japan). For each species, densitometric values at each time point were normalized to their maximal value during the time course.

### Mathematical modeling

The description using ordinary differential equations of the reaction pathway for bovine APC inactivation of bovine FVa reported by Hockin et al. [33] was used as a starting framework (Figure 2; Table 1). The scheme describes a random order cleavage process for Arg^306^ and Arg^506^ in which the cleavages are independent of each other and characterized by substantially different rate constants. Our simulator employs a fourth order Runga-Kutta algorithm to generate a series of time-dependent concentration profiles for all reactants, intermediates, and products.

Factor Va cofactor activity will be represented by the cumulative presence of FVa^HC^ species present at any point in time. For measurements of *prothrombinase* activity, the sum of all FVa species was added to the weighted (20 %) sum of FVa_i_^306^, FVa_i_^506^, and FVa_i_^306/506^. Factor Va inactivation fragments will be represented by including respective fragments both associated and dissociated from the FVa^LC^.

To generate an appropriate simulation of the empirical experiment where FXa and FVa are pre-incubated together on the phospholipid surface to form the *prothrombinase* complex prior to the addition of APC, the concentrations of free FXa and FVa, and the *prothrombinase* complex were solved. For example, studies with 200 pM FVa and 100 pM FXa, for each potential K_D_ (e.g. 0.1 nM or 0.5 nM, respectively), the values of the three initial conditions ranged from 41–74 pM for free FXa, 141–174 pM for free FVa, and 59–26 pM for *prothrombinase*. The concentrations of the three species then served as the initial conditions in the simulations in conjunction with either APC concentration.

*In silico* simulations were carried out with the initial concentrations used in the empirical experiments and the relevant output calculated and compared to the appropriate analyte measured in the empirical experiments.

## Abbreviations

APC: Activated protein C; EDTA: (Ethylene-dinitrilo) tetraacetic acid; FVa: Activated factor V; FVaHC: Heavy chain of FVa; FVaLC: Light chain of FVa; FVai: A two chain molecule composed of the FVaLC and FVa1-306 fragment; FXa: Activated factor X; FXa*: Active site-blocked activated factor X; HEPES: N-[2-Hydroxyethyl]piperazine-N’-2-ethanesulfonic acid; HBS: 20 mM HEPES, 150 mM NaCl, pH 7.4; min: minutes; PC: Protein C; PC:PS vesicles: Single bilayer phospholipid vesicles composed of 75 % 1,2-dioleoyl-sn-glycero-3-phosphocholine and 25 % 1,2-dioleoyl-sn-3-glycero-3-[phospho-L-serine]; PEG: Polyethylene glycol, average molecular weight = 8000; PT: Prothrombin; RMV: Relative to maximal value; s: Seconds; TF: Tissue factor.

## Competing interests

Ms. Maria Cristina Bravo, Dr. Thomas Orfeo, and Dr. Stephen J. Everse declare no conflict of interest. Dr. Kenneth G. Mann is Chairman of the Board of Haematologic Technologies, Essex, VT where some of the proteins were purchased for these investigations.

## Authors’ contributions

MCB designed/performed research, analyzed data and wrote the first draft of the paper. TO, KGM and SJE contributed to experimental design and to the writing of the paper. All authors read and approved the final manuscript.
